# Characterization of Irradiation Crosslinked Polyamides for Solar Thermal Applications—Basic Thermo-Analytical and Mechanical Properties

**DOI:** 10.3390/polym10090969

**Published:** 2018-09-01

**Authors:** Patrick R. Bradler, Joerg Fischer, Gernot M. Wallner, Reinhold W. Lang

**Affiliations:** Institute of Polymeric Materials and Testing, Johannes Kepler University, 4040 Linz, Austria; joerg.fischer@jku.at (J.F.); gernot.wallner@jku.at (G.M.W.); solpol@jku.at (R.W.L.)

**Keywords:** polyamide 66, short glass fiber reinforced polyamide 66, irradiation crosslinking, tensile behavior, effect of water

## Abstract

Polyamide 66 (PA 66) and short glass fiber reinforced versions of PA 66 are widely used for solar-thermal applications, in which thermal and environmental loading of components is from high importance. In this study, the influence of crosslinking via electronic beam irradiation on the morphology and mechanical behavior of unreinforced PA 66 and two types of short glass fiber reinforced PA 66 (30 wt % glass fiber content, 35 wt % glass fiber content) was investigated. In total, five different electronic beam irradiation doses in the range of 0 and 200 kGy were applied. Besides experiments with unconditioned specimens, also preconditioned specimens saturated with water at 80 °C for seven days were investigated. It was found that irradiation causes a shift to lower melting temperatures and lower melting enthalpies, while simultaneously leading to higher glass transition temperatures (*T*_G_), increasing small strain modulus values and higher tensile strengths. Also, as expected, preconditioning samples in water at 80 °C to water uptake saturation leads to a shift to lower *T*_G_ values (‘plasticization’ effect). In terms of tensile behavior at room temperature, water saturated specimens (being above *T*_G_ at room temperature) exhibited lower modulus and tensile strength values compared to quasi-dry specimens (being below *T*_G_ at room temperature).

## 1. Introduction

In a wide range of applications for polyamides (PA), thermal and environmental loading of components is of high importance with correspondingly high requirements on the material performance profiles [1,2]. In many instances, therefore glass fiber reinforced versions of PA are the material of choice, which is also the case for the solar-thermal integrated collector storage (ICS) system by GREENoneTEC Solarindustrie GmbH (St. Veit/Glan, Austria) for hot water preparation [3]. The components typically have to withstand significant mechanical loading (e.g., internal pressure) superimposed by specific environmental conditions (e.g., exposure to water and elevated temperature). Electronic beam radiation of PA provides a good opportunity for modification, levelling up the technical polymers to the properties of high performance plastics [4,5]. The opportunity to significantly enhance certain performance properties of plastics by irradiation crosslinking has long been known, starting with investigations on crosslinking of polyolefines [6,7,8], subsequently followed by investigations for polyamides [9,10,11,12,13]. Thus, it was found that irradiation crosslinking of thermoplastics mainly occurs in the amorphous regions of the polymer [14,15]. Moreover, specific crosslinking additives may act to support the crosslinking mechanism [4].

Previous research work performed in our laboratory was carried out to investigate the long-term behavior of uncrosslinked polyamides and polyphthalamides for various solar energy applications (e.g., solar thermal and PV collectors) [16,17]. The main aim of the present paper is to take a closer look on the behavior of irradiation crosslinked versions of PA 66 under quasi-dry and water saturated conditions. The present paper describes the influence of the irradiation dose on basic thermo-analytical characteristics (investigated by differential scanning calorimetry) and on thermo-mechanical properties (involving dynamic mechanical analysis and tensile tests). Thus, this paper should investigate the influence of different irradiation doses under application near conditions assessing the effect of crosslinking as a possibility to local improvement of PA-grades and examine the potential in solar energy applications.

Irradiation crosslinking of polymers is based on triggering various chemical crosslinking reactions by exposure of the polymer to a sufficiently high irradiation energy in terms of irradiation intensity and time [18]. Thus, it is well known that irradiation-crosslinking may yield improved mechanical and thermal properties particularly under superimposed environmental exposure [4,14,19]. Frequently reported effects of irradiation-crosslinking of semi-crystalline plastics are higher modulus values particularly above the polymer glass transition temperature *T*_G_, an effect that is caused by reduced molecular segmental mobility in the amorphous regions governed by the formation of covalent bonds. Frequently used is an electronic beam irradiation in which the energy dose ‘*D*’ in Gray (*G*y) is the most important parameter. In Equation (1), ‘*D*’ depends on ‘d*E*’ in Joules (J), which is the radiation energy, and ‘dm’ is the mass in kilogram (kg) related to the irradiated volume element.

*D* = d*E*/d*m* (1)

**Figure 1:**
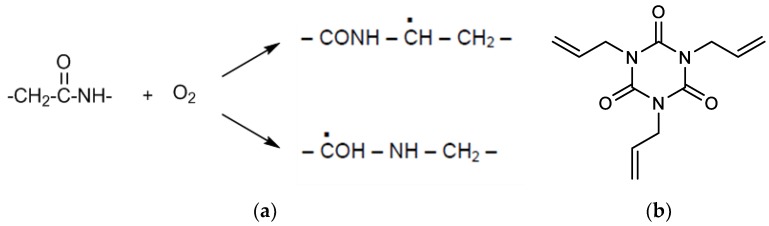
(**a**) Schematic illustration of the radical formation for a polyamide and (**b**) the chemical structure of the irradiation additive TAIC.

**Figure 2:**
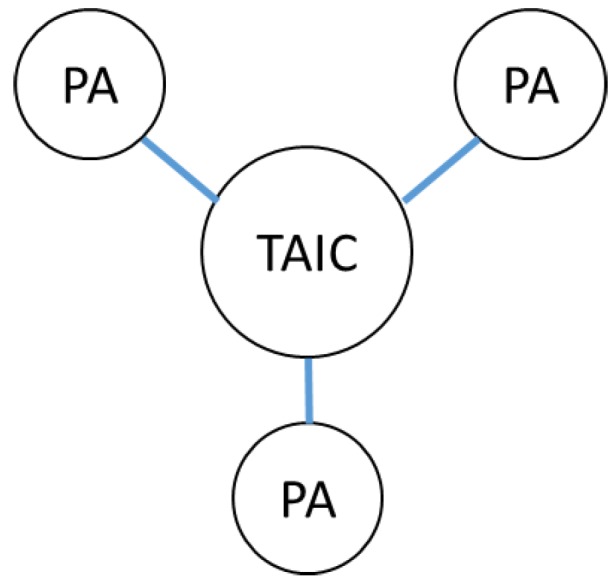
Final structure of the crosslinked PA and TAIC.

The crosslinking reaction starts with the formation of radicals due to the irradiation energy. This reaction mechanism is based on the elimination of specific chain atoms via an irradiation with an electronic beam. In the case of polyamides, mainly the hydrogen atom is eliminated. There are different mechanisms of radical formation as a precondition for subsequent crosslinking reactions, two of them are schematically illustrated in Figure 1a [20]. Additionally, the cross-linkable PAs are typically compounded with the chemical substance 3,5-Triallyl-I,3,5-triazin-2′4,6(1*H*,3*H*′5*H*)-trion, known as Triallyl Isocyanurate or TAIC (see Figure 1b). The irradiation induced polymer radical may react with the crosslinking agent (TAIC), creating a three-dimensional network of polymer chains. The resulting structure after the irradiation crosslinking process between PA and TAIC is schematically depicted in Figure 2.

## 2. Materials and Methods

Three different commercially available polyamide (PA) grades containing crosslinking additives were investigated. The TAIC content for all grades was below 5 wt %. While one PA is an unreinforced grade, the other two are reinforced with 30 and 35 wt % short glass fibers. The designation of the materials along with information on the supplier are listed in Table 1.

In a first step, these materials were injection molded to standardized multi-purpose specimens (tensile bars according to ISO 527-2) [21]. One set of specimens for each of these materials were kept and used to represent the reference material state in the unirradiated and non-water exposed condition. A second set of specimens, representing the majority of specimens produced were exposed to an irradiation energy of 50, 100, 150, and 200 kGy, respectively. The irradiation was carried out at the company Mediscan GmbH Co KG (Kremsmünster, Austria) using an irradiation device of the type Rhodotron TT-100 (IBA International, Louvain-La-Neuve, Belgium). This device uses a scanning magnet at a frequency of 100 Hz and a 90° vertical bending magnet operating at 10 MeV with a beam power of 40 kW.

As to the influence of water exposure, a series of specimens for each material were preconditioned to water uptake saturation levels by water immersion for seven days at 80 °C in deionized water prior to testing. Throughout this paper, the water saturated state is referred to as ‘preconditioned’ material state, and the water uptake saturation levels were recorded for the various material grades and states. All non-water exposed specimen states are referred to as ‘quasi-dry’ or ‘as-received’ condition. Subsequent weight loss measurements upon elevated temperature exposure indicated a water content of about 0.5 to 0.9 wt % for the as-received condition. While the as-received state represents the upper-bound environmental condition under dry environmental conditions, the preconditioned state represents the lower bound, as the properties of PA is strongly influenced by the water content. Both states are potential environmental conditional in solar energy applications.

While the injection molded ISO 527-2 multi-purpose specimens were used directly for tensile testing, samples and specimens and for the differential scanning calorimetry (DSC) investigations and the dynamic mechanical analysis (DMA) were taken from the center-portion of the multi-purpose specimens.

DSC tests were carried out on a Perkin Elmer DSC, type 8500 (Perkin Elmer Inc., Waltham, MA, USA) with nitrogen as purge gas and a flow rate of 20 mL/min, and using a sample weight of about 8 mg. For testing, this sample was encapsulated in an aluminum pan. The procedure consisted of a first heating, subsequent cooling, and a second heating phase, each in the temperature range of −30 °C to 270 °C with a constant heating/cooling rate of 10 K/min. The DSC measurements were accomplished to determine the melting behavior, where the melting peak in the first heat-up phase represents the processing history of injection molding, and the melting peak in the second heat-up phase is characteristic for the semi-crystallinity achieved under controlled cooling in the DSC device. For each material state, two samples were investigated. While there was excellent reproducibility between the two samples, mean values are reported below.

DMA investigations were performed with an Anton Paar Physica MCR 502 rheometer (Anton Paar GmbH, Graz, Austria) in a sinusoidal torsion loading mode to determine the storage modulus *G*’ and the loss factor tan δ as a function of temperature. The tests were performed in the temperature range from −75 to 175 °C with a constant heating rate of 2 K/min using nitrogen as surrounding gas. The loading was set constant with an angular displacement of 0.05% and a loading frequency of 1 Hz [22].

Tensile tests on multi-purpose specimens according ISO 527-1 were carried out on a universal testing machine of the type Z020 (Zwick GmbH & Co., KG, Ulm, Germany). The testing speed was 1 mm/min for the Young’s moduli measurement (strain range of 0.05% and 0.25%; strain determination with a multiXtens extensometer also supplied by Zwick GmbH & Co., KG, Ulm, Germany). At 0.25% strain, the testing speed was altered to 5 mm/min until ultimate failure for all specimens (strain determination via crosshead displacement in this regime).

## 3. Results and Discussion

**Figure 3:**
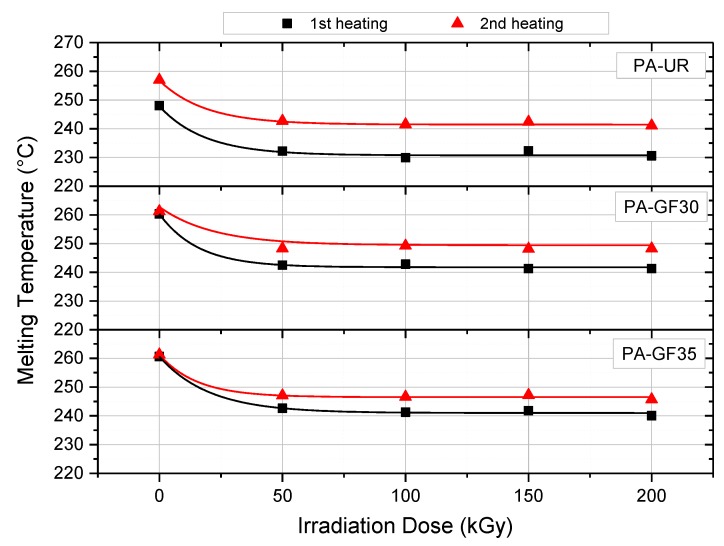
Melting peak temperature *T*_m,p_ as a function of irradiation dose of three different PA grades for the first and second heating run.

The melting peak temperatures (*T*_m,p_), obtained from DSC experiments (first and second heating) are depicted as a function of the electronic beam irradiation doses in Figure 3. For all three PA grades, *T*_m,p_ values are shifted to lower temperatures with increasing irradiation dose up to about 100 kGy, than remaining constant at higher irradiation doses. The initial drop in *T*_m,p_ values is quite significant and amounts to about 20 K. The decrease of *T*_m,p_ can be caused by an increasing irregularity in the crystalline phase due to electronic beam irradiation. Apart from the unirradiated states of PA-GF30 and PA-GF35, where differences were negligible, the melting peak temperatures of the second heating run were found to be up to 10 K higher than in the first heating. In agreement to studies by others [13], the difference in *T*_m,p_ values between first and second heating is most pronounced for the PA-UR and reduces with increasing glass fiber content.

**Figure 4:**
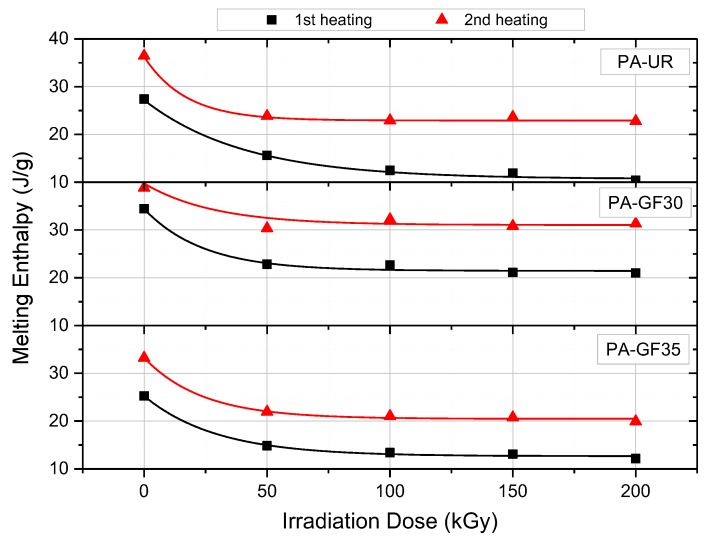
Melting enthalpy as a function of irradiation dose of three different PA grades for the first and second heating run.

The melting enthalpy for all three investigated materials is plotted in Figure 4. As a function of the irradiation dose, again for the first and second heating run. As with the melting peak temperature values, a decrease of the melting enthalpy with increasing irradiation dose is observed, at least up to about 100 to 150 kGy. As expected, a lower melting enthalpy was found for the reinforced PA grades due to the lower amount of polymer matrix material in the sample. For the second heating run, higher enthalpy values were found, resembling a similar tendency observed for the melting temperatures. When assuming an enthalpy of 230 J/g for 100% crystalline PA [23], a decrease in the degree of crystallinity of about 6% can be observed between the unirradiated and 200 kGy irradiated samples.

**Figure 5:**
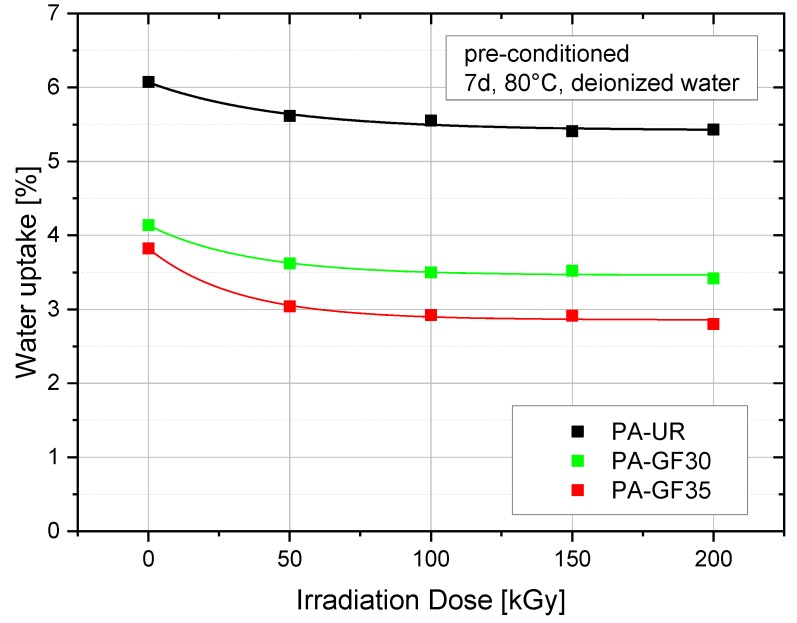
Water uptake as a function of irradiation dose of three different PA grades.

The water uptake at water saturation level (exposure time of seven days at 80 °C [16]) of the ISO-527-2 multi-purpose specimens is depicted in Figure 5 as a function of irradiation dose. The highest water uptake was found for unirradiated PA-UR, amounting a weight gain of about 6%. Expectedly, for the reinforced grades PA-GF30 and PA-GF35 a lower moisture uptake is observed due to a lower amount of water absorbing PA matrix material. Within each grade, a decrease of water uptake was found up to an irradiation dose of 100 kGy, essentially remaining constant for higher irradiation doses (at least up to 200 kGy). Apparently, there is a good correlation on the influence of irradiation dose on the semi-crystalline morphology as characterized by the melting peak temperatures and the melting enthalpy, on the one hand, and the saturation water uptake, on the other. Thus, these results corroborate the frequent assertion that irradiation acts primarily to crosslink the amorphous regimes [14,15] which also are considered to control water absorption [24,25].

**Figure 6:**
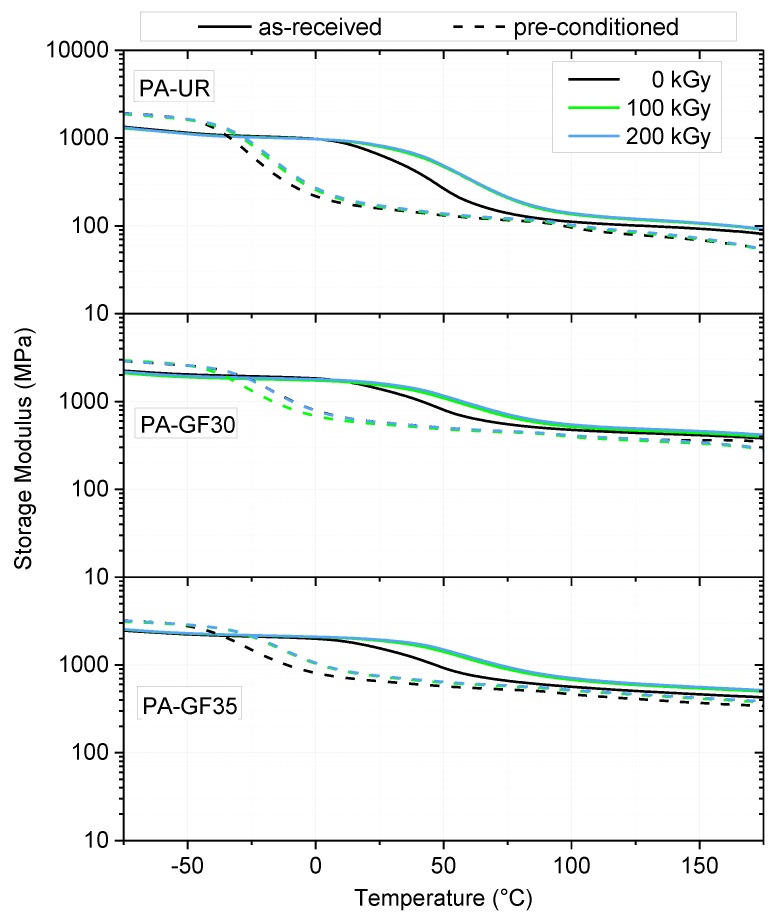
DMA curves for the three PA grades depicting the effect of water absorption and irradiation dose.

Figure 6 depicts the DMA curves for as-received and water preconditioned specimens in terms of the dependency of storage modulus on temperature. As pointed out above, in all cases and material irradiation states water absorption leads to a decrease in the glass transition temperature *T*_G_ (plasticization effect), while the storage modulus values of water saturated materials below *T*_G_ are higher than those of corresponding materials in the as-received state (anti-plasticization effect) [26]. On the other hand, irradiation clearly enhances the *T*_G_ values compared to the unirradiated states. Furthermore, the modulus in the rubbery plateau (above *T*_G_) is improved with increased irradiation dose. This effect can be reasoned by the lower mobility in the polymer with increasing crosslinking density, but again, the difference between materials irradiated with 100 kGy compared to 200 kGy seems to be quite negligible.

**Figure 7:**
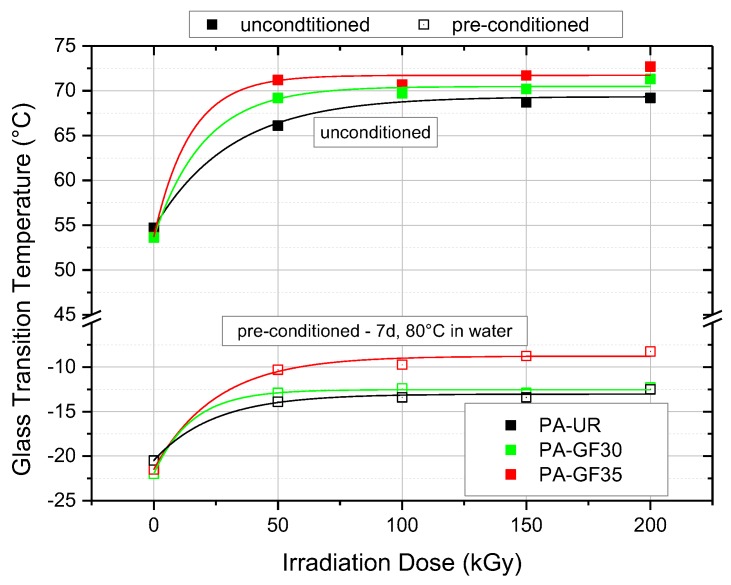
Glass transition temperatures for the three PA grades depicting the effect of water absorption and irradiation dose.

The quantitative influence of irradiation and water uptake on the *T*_G_ values is depicted in Figure 7. The effect of water absorption in the unirradiated state is to decrease the *T*_G_ from about 54 °C to about −22 °C. Irradiation in all cases leads to higher *T*_G_ values of about 70 °C in the as-received state and about −10 °C in the water saturated state.

**Figure 8:**
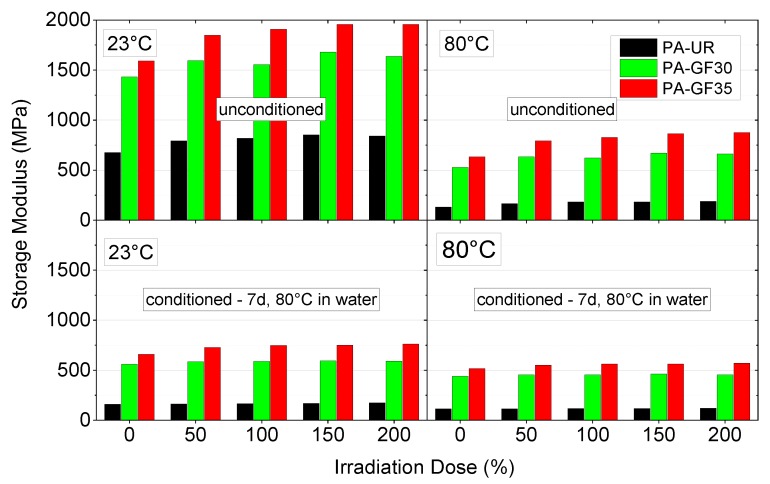
Storage modulus at 23 °C and 80 °C for the three PA grades depicting the effect of water absorption and irradiation dose.

Analogously, quantitative results for the effect of irradiation and water uptake on storage modulus values *G*’ are shown in Figure 8 as bar charts for two temperatures, 23 and 80 °C, respectively. To start with, and as expected, in all material states and for all test conditions modulus values are seen to increase significantly with the glass fiber content from about 71% up to about 86% compared to PA-UR. The influence of water uptake in the temperature range from 23 to 80 °C is dominated by the plasticization effect, thus leading to a reduction in modulus values for water saturated specimens compared to as-received samples. The effect is more pronounced for a test temperature of 23 °C, as the as-received samples and the water saturated samples are below and above their *T*_G_ values, respectively, at this temperature. Finally, and equivalent to the findings for the dependence of melting temperature, melting enthalpy, moisture uptake and glass transition temperature on irradiation, increasing the irradiation dose results in a corresponding increase in modulus values up to about 100 kGy, with only minor additional effects for higher irradiation values. For example, in the as-received condition, irradiation was found to increase modulus values by about 25%, 17%, and 23% for PA-UR, PA-GF30, and PA-GF35, respectively.

**Figure 9:**
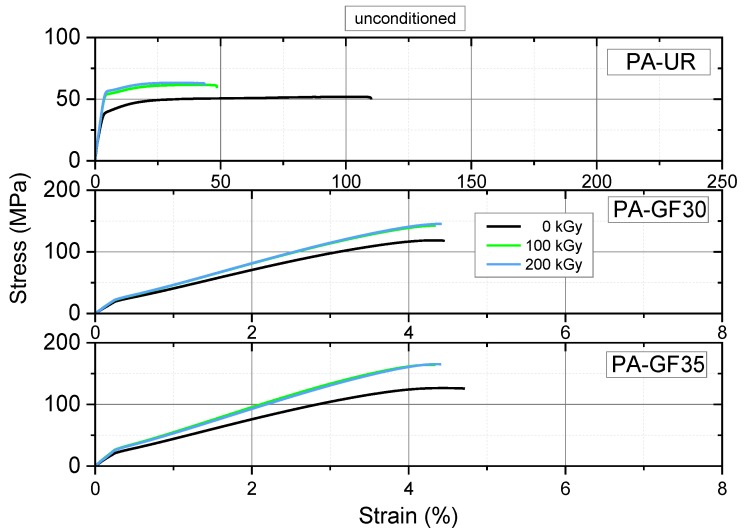
Tensile stress–strain curves for three PA grades in the as-received condition at 23 °C depicting the effect of irradiation dose.

**Figure 10:**
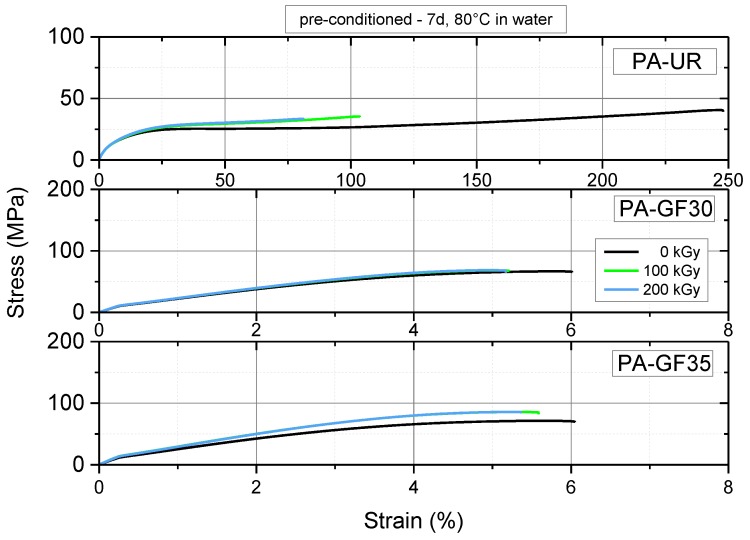
Tensile stress–strain curves for three PA grades in the water saturated condition at 23 °C depicting the effect of irradiation dose.

The tensile behavior of the materials investigated is illustrated depicting typical stress–strain curves in Figure 9 and Figure 10 for as-received and water saturated specimens, respectively. Please note the difference scales on the strain axis for unreinforced and glass fiber reinforced PAs. The main effects of irradiation in the as-received condition of unreinforced PA-UR are an increase in yield stress from about 39 to 57 MPa accompanied by significant reduction in nominal strain-at-break from about 110% to less than 50% (Figure 9 top). There are no significant differences between 100 and 200 kGy irradiation dose. Adding 30 to 35 wt % glass fibers to PA 66 increases the maximal tensile stress levels to above 100 MPa, with material states exposed to 100 and 200 kGy, respectively, again being equivalent but exhibiting some 22% to 30% higher values than unirradiated materials. However, contrary to unreinforced PAs, the effect of irradiation on strain-at-break compared to unirradiated material states diminishes (Figure 9, middle and bottom). This is certainly related to the fact that the addition of short glass fibers leads to a significant reduction in the strain-at-break to about 5% compared to more than 100% for PA-UR. Moreover, ultimate failure in short glass fiber reinforced polymers is known to be governed by a variety of mechanisms including matrix strain magnification, fiber debonding, and fiber pull-out not available in neat matrix systems [27]. Similar tendencies were observed for the tensile behavior of these materials in the water saturated state (Figure 10, however with generally enhanced values for strain-at-break and reduced values for maximum tensile stresses due to the above-mentioned plasticization effect of water in PAs.

**Figure 11:**
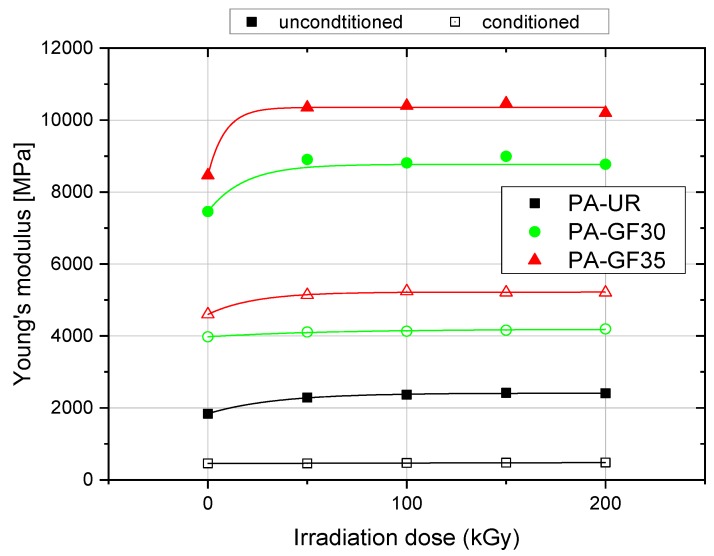
Young’s modulus at 23 °C for the three PA grades depicting the effect of water absorption and irradiation dose.

Since many structural components also in solar-thermal applications are designed for stiffness, the effect of irradiation dose on Young’s modulus at 23 °C is shown in Figure 11 for the three material grades investigated under as-received and water saturated conditioned. In the as-received material states (i.e., all materials tested below *T*_G_), a pronounced increase in Young’s modulus is seen up to an irradiation dose of about 100 kGy with only minor additional effects at higher irradiation levels. The modulus enhancements by irradiation are in the range of 20% to 36% compared to the unirradiated material states. In the water saturated material states, the influence of irradiation is significantly reduced as all materials at 23 °C are now above their *T*_G_ regimes. Here, the maximum modulus enhancements are limited to about 15%. Selected properties as Young’s moduli, strain-at-break values, and maximum stresses for unconditioned and conditioned specimens are summarized in Table 2.

## 4. Conclusions

In this study, the effect of crosslinking unreinforced and short glass fiber reinforced grades of PA 66 by electronic beam irradiation on the morphology and the thermo-mechanical and tensile properties was investigated. An irradiation dose of up to about 100 kGy, which corresponds to the irradiation intensity recommended by the material supplier, leads to enhancements in the glass transition temperature, Young’s modulus and torsional modulus, and maximum tensile stress values. Conversely, over the same range of irradiation, values for the melting temperature, the melt enthalpy, water uptake at saturation, and strain-at-break are reduced. Above the irradiation level of about 100 kGy, no further significant effects on these properties are observed. Any effects of water conditioning on material properties found in unirradiated PAs are also present in irradiation crosslinked PAs, however to a diminishing extent. While from a design for enhanced stiffness perspective, irradiation is certainly advantageous, a component failure perspective needs to be also account for the long-term effects of irradiation crosslinking on the mechanical failure behavior of these materials, an aspect which is the subject of Part II of this paper series.

## Figures and Tables

**Table 1:** Materials investigated and material supplier

Material Designation	Glass Fiber Content	Manufacturer
PA-UR	0 wt %	PTS Plastic-Technologie-Service, Marketing und Vertriebs GmbH
PA-GF30	30 wt %	Nilit Plastics Europe GmbH
PA-GF35	35 wt %	PTS Plastic-Technologie-Service, Marketing und Vertriebs GmbH

**Table 2:** Overview of the tensile test data for the unconditioned and conditioned specimens

Material State:		Unconditioned ^1^			Conditioned ^2^		
Material Designation	Irradiation Dose in kGy	Young’s Modulus in MPa	Strain-at-Break in %	Maximum Stress in MPa	Young’s Modulus in MPa	Strain-at-Break in %	Maximum Stress in MPa
PA-UR ^3^	0	1840 ± 90	96 ± 12	52 ± 0.1	461 ± 4	233 ± 21	39 ± 3
	50	2280 ± 20	50 ± 3	59 ± 0.4	456 ± 2	126 ± 6	36 ± 2
	100	2370 ± 20	47 ± 2	61 ± 0.4	469 ± 2	110 ± 9	36 ± 1
	150	2420 ± 20	41 ± 4	63 ± 0.4	476 ± 8	94 ± 10	34 ± 2
	200	2410 ± 10	41 ± 2	63 ± 0.1	482 ± 1	82 ± 2	33 ± 1
PA-GF30 ^4^	0	7460 ± 120	4.5 ± 0.1	118 ± 1	3980 ± 30	6.1 ± 0.1	67 ± 1
	50	8900 ± 115	4.4 ± 0.1	141 ± 1	4110 ± 20	5.3 ± 0.1	67 ± 1
	100	8810 ± 250	4.4 ± 0.1	143 ± 1	4130 ± 50	5.3 ± 0.1	68 ± 1
	150	9000 ± 180	4.4 ± 0.1	145 ± 1	4160 ± 50	5.3 ± 0.1	68 ± 1
	200	8770 ± 80	4.4 ± 0.1	145 ± 1	4200 ± 30	5.2 ± 0.1	68 ± 1
PA-GF35 ^5^	0	8470 ± 280	4.7 ± 0.1	129 ± 2	4600 ± 230	6.0 ± 0.1	73 ± 3
	50	10,350 ± 450	4.5 ± 0.1	158 ± 2	5140 ± 20	5.8 ± 0.1	84 ± 1
	100	10,400 ± 360	4.3 ± 0.1	163 ± 1	5250 ± 40	5.6 ± 0.1	85 ± 1
	150	10,470 ± 250	4.7 ± 0.1	166 ± 3	5210 ± 60	5.6 ± 0.1	86 ± 1
	200	10,200 ± 170	4.4 ± 0.1	165 ± 1	5200 ± 30	5.5 ± 0.2	86 ± 1

^1^ stored at room temperature; ^2^ immersed seven days at 80 °C in deionized water prior to testing; ^3^ unreinforced polyamide; ^4^ polyamide with 30 wt % glass fiber reinforcement, ^5^ polyamide with 35 wt % glass fiber reinforcement.
